# The Effect of Music-Based Intervention on General Cognitive and Executive Functions, and Episodic Memory in People with Mild Cognitive Impairment and Dementia: A Systematic Review and Meta-Analysis of Recent Randomized Controlled Trials

**DOI:** 10.3390/healthcare10081462

**Published:** 2022-08-03

**Authors:** Erika Ito, Rui Nouchi, Jerome Dinet, Chia-Hsiung Cheng, Bettina Sandgathe Husebø

**Affiliations:** 1 School of Medicine, Tohoku University, Sendai 980-8575, Japan; itoeri@hotmail.co.jp; 2 Department of Cognitive Health Science, Institute of Development, Aging, and Cancer, Tohoku University, Sendai 980-8575, Japan; 3 2LPN (Laboratoire Lorrain de Psychologie et Neurosciences de la Dynamique des Comportements), Université de Lorraine, F-54000 Nancy, France; jerome.dinet@univ-lorraine.fr; 4 Department of Occupational Therapy and Graduate Institute of Behavioral Sciences, Chang Gung University, Taoyuan 33302, Taiwan; ch.cheng@mail.cgu.edu.tw; 5 Healthy Aging Research Center, Chang Gung University, Taoyuan 33302, Taiwan; 6 Centre for Elderly and Nursing Home Medicine, Department of Global Public Health and Primary Care, Faculty of Medicine, University of Bergen, 5009 Bergen, Norway; bettina.husebo@uib.no

**Keywords:** music intervention, music therapy, dementia, MCI, nonpharmacological therapy, cognitive functions

## Abstract

Background: Music-based intervention has been used as first-line non-pharmacological treatment to improve cognitive function for people with mild cognitive impairment (MCI) or dementia in clinical practice. However, evidence regarding the effect of music-based intervention on general cognitive function as well as subdomains of cognitive functions in these individuals is scarce. Objective: To evaluate the efficacy of music-based interventions on a wide range of cognitive functions in people with MCI or dementia. Method: We searched the effect of various music therapies using randomized controlled trials on cognitive function using several databases. Studies based on any type of dementia or MCI were combined. The effects of music-based intervention on each cognitive function were pooled by meta-analysis. Results: A total of 19 studies involving *n* = 1024 participants (mean age ranged from 60 to 87 years old) were included. We found statistically significant improvements in MMSE (general cognitive function), the Frontal Assessment Battery (executive function), and the Auditory Verbal Learning Test (episodic memory). Conclusions: This study provides positive evidence to support music-based interventions for improving a wide range of cognitive functions in older adults with MCI and dementia. Therefore, we recommend increased use of music in people’s homes, day care centers and nursing homes. This study was registered with PROSPERO, number 250383.

## 1. Introduction

The number of people with dementia and mild cognitive impairment (MCI) has rapidly increased in recent decades and related challenges have become a global burden [1]. Dementia and MCI are characterized by irreversible impairment of cognitive function, behavioral and psychological symptoms of dementia, and decrease in the activities of daily living (ADL) [2]. Decreased cognitive function is one of the main reasons why the lives of people with dementia become difficult [3]. In people with dementia and MCI, cognitive decline also negatively affects quality of life (QoL), ADL functioning, and communication with relatives and healthcare professionals [4]. Therefore, it is important for cognitive function to be improved in the daily lives of people with dementia and MCI.

Pharmacological and non-pharmacological intervention are two approaches for improving cognitive function. The latest pharmacological approaches indicate the beneficial effects of acetylcholinesterase inhibitors and memantine in people with Alzheimer’s disease [5]. However, the costs and risks associated with utilizing pharmacological therapy have been an issue [5]. Additionally, antidepressants, sedatives, and anti-dementia drugs are commonly used as add-on therapies, but have shown negative impacts on QoL [6]. Due to fewer risks and adverse effects, a non-pharmacological approach using cognitive activities, physical exercise, and art and play therapies has been suggested as a first line treatment strategy for the neuropsychiatric symptoms of dementia [6]. Previous studies have shown that these intervention programs lead to improvements in cognitive function in people with dementia and MCI [7,8]. Thus, non-pharmacological approaches have attracted the attention of aging researchers and caregivers.

Musical skill is a preserved skill in people with dementia [9,10]. Consequently, music-based intervention is a major strategy of the non-pharmacological intervention approach [11]. Music-based intervention is defined as any intervention using “music” (music as a cultural product usually involving some combination of melody, rhythm and harmony cognitively processed by the human brain) to study the therapeutic effect [11]. “Music” is then differentiated from single pitch sound and sound (or aspects of music) transduced into vibrotactile sensation of neuromodulatory and physiological effects [12,13]. The review in this paper focuses exclusively on music-based interventions which were mainly categorized into music therapy by a trained music therapist and music medicine by healthcare or music professionals [11,14]. At the beginning, music therapy was defined as “an intentional use of properties and the potential of music and its impact on the human being” [15]. Recently, the American Music Therapy Association has narrowed this definition to: “Music Therapy is the clinical and evidence-based use of music interventions to accomplish individualized goals within a therapeutic relationship by a credentialed professional who has completed an approved music therapy program”(https://www.musictherapy.org/about/musictherapy/, access date: 4 July 2022). It suggests that music therapy involves therapeutic relationships between participants and music therapists. On the other hand, music medicine does not need these relationships.

Music has been widely used in clinical practice throughout history [16]. However, the results of studies on the effectiveness of music-based intervention lack consistency. Some meta analyses focusing on global cognitive function showed that music-based intervention had small to moderate effects on maintaining or improving cognitive function [17,18]. Other meta-analyses showed inconsistent results in that music-based intervention was found to have no effect on impairment [19]. Due to the small number of studies included in the previous meta-analyses and these inconsistent results [18,19,20], it is difficult to conclude whether music-based intervention would have positive effects on cognition in aging populations or not. It still needs to be investigated whether music-based intervention has the potential to improve cognitive function in people with dementia and MCI.

Previous meta-analysis studies mainly focused on the benefits of music-based intervention for general cognitive function measured by the Mini Mental State Examination (MMSE). However, prior evidence suggests that cognitive function has multiple subdomains [21]. In addition, each individual with dementia shows deterioration of cognitive function in a different way. In terms of the subdomains of cognitive function, some show specific deterioration of memory while others show decline in executive function [21]. Therefore, it is essential to investigate whether music-based intervention would have positive effects on each cognitive function subdomain. In this study, we performed a systematic review and meta-analysis to evaluate the effectiveness of music-based intervention on each subdomain of cognitive function in people with dementia and MCI.

## 2. Materials and Methods

Analysis methods and eligibility criteria were specified in advance and documented in a protocol registered on PROSPERO (International Prospective Register of Systematic Reviews; Record ID = 250383). This systematic review and meta-analysis were conducted in accordance with the PRISMA check-list [22]. Please see Supplementary Materials (Supplemental Table S1).

### 2.1. Eligibility Criteria

Trials were considered for inclusion in this review if the criteria set out below were met.

**Participants**: Men and women aged 60+ with a clinical diagnosis of cognitive impairment or dementia. **Intervention**: Any music-based intervention or community music activity including listening to music, singing, playing an instrument, and music with movement or exercise. The design of the trial must have been such that the independent effects of either exercise, cognitive, or dual-task training on cognition could be analyzed. We delimited our review to exclude interventions focusing on music or sound as vibrotactile stimulation, single frequency sound, or sound for its vibratory effect. **Comparison**: Any concurrent control group was eligible, including no intervention/usual care, meditation, pharmacological intervention, exercise intervention, late intervention, and painting or other art related activities. **Outcome**: Any validated cognitive tests reported at the baseline and follow up after exposure to any type of music-based intervention. **Study design**: Randomized controlled trials that allocated individuals to either an intervention or concurrent control group.

### 2.2. Search Strategy

We searched scientific articles which were published between January 2000 and April 2021. The following electronic databases were used to search for completed trials: PubMed, CINAHL, PsycINFO, and Google Scholar. For the PubMed search, we checked the “randomized controlled trial” box. For the other searches, we added “randomized controlled trial” as a key word in addition to certain other keywords. For the cognitive function domain, keywords included (“music” OR “sing” OR “song” OR “listen”) AND (“dementia” OR “elderly” OR “Alzheimer’s” OR “cognitive impairment”) AND (“cognitive function” OR “cognition” OR “memory” OR “attention” OR “executive function” OR “processing speed”). All key search terms were combined, where possible, with medical sub-headings (MeSH) and indexed terms to identify potentially relevant studies. Trials on interactive music intervention and listening to music were manually identified from the title and abstract previews of all search records.

### 2.3. Inclusion and Exclusion Criteria

Based on the previous studies [23,24,25,26], we set the inclusion and exclusion criteria. Randomized controlled trials were included if they met the following inclusion criteria: (1) participants with clinical diagnosis of dementia or cognitive impairment. We also included persons with Alzheimer’s disease, Parkinson’s disease, and mixed dementia; (2) studies that investigated the treatment effect of any type of music-based intervention; (3) studies that reported at least one of the following outcomes: general cognitive function, episodic memory, working memory, attention, processing speed, and executive function; (4) studies that measured the scores of the clinical assessment scales from baseline to endpoint or follow up among the intervention and control groups. Studies were excluded if the study design was not a randomized controlled trial, they compared participants with dementia/MCI and healthy individuals or only included healthy adults, or there were insufficient details from which the study outcomes could be derived.

### 2.4. Study Outcomes

Based on the previous studies [25,26], cognitive function was categorized into seven different cognitive domains: general cognitive function, episodic memory, working memory, short-term memory, attention, processing speed, and executive function. We used all of the available clinical cognitive assessment scales in each domain from the included studies as our primary outcomes. Examples of the primary outcomes of this study are the change scores in MMSE [27], Severe Impairment Battery (SIB) [28], Montreal Cognitive Assessment (MoCA) [29], Auditory Verbal Learning Test (AVLT) [30], The Word Memory Test (WMT) [31], Trail Making Test A (TMT-A) [32], Digit Symbol Substitution (DSST) [33], Verbal Fluency Test, Trail Making Test B (TMT-B) [32] and Frontal Assessment Battery (FAB) [34]. The MMSE, SBI, and MoCA focused on general cognitive function; the AVLT and WMT focused on episodic memory and shortterm memory, respectively; the TMT-A and DSST focused on processing speed; and the Verbal Fluency Test, TMT-B, and FAB focused on executive function.

Standard mean difference (SMD) with standard deviation (SD) or 95% confidence interval (CI) was used to evaluate the changes in assessment scores between the music-based intervention and control groups. For the TMT-A and TMT-B, since a longer time indicated a lower cognitive function [32], we reversed the score for analysis. A higher score for the MMSE, SBI, MoCA, AVLT, WMT, DSST, FAB, TMT-A, and TMT-B indicated better cognitive function.

### 2.5. Data Extraction

Two investigators (E.I. and R.N.) independently assessed the relevance of the search results and abstracted the demographic details of individual trials into a data extraction Excel form, including year of publication, study location, number of participants included, mean age, number and percentage of female participants, stages of dementia, recruitment site, type of music-based intervention, control group intervention, treatment period and duration, and all clinical assessment scales.

### 2.6. Risk of Bias Assessment

E.I. and R.N. independently assessed the quality of each study according to the methodology section of the Consolidated Standards of Reporting Trials (CONSORT) statement [35]. A 14-point scale was designed for the evaluation of study quality including (1) random allocation, (2) treatment allocation concealed, (3) groups/subjects similar at baseline regarding important prognostic values, (4) eligibility criteria specified, (5) blinded outcome assessor, (6) blinded care provider, (7) blinded patients/people, (8) point estimates and measures of variability presented for the primary outcome measures, (9) intention-to-treat analysis, (10) details of random allocation methods, (11) adequate description of the control/comparison group, (12) between-group statistical comparison, (13) reporting dropouts, and (14) reporting CONSORT statement.

The potential risk of bias [36] for included trials was checked using the Cochrane Risk of Bias assessment tool with the following domains: (1) random sequence generation of intervention and control groups, (2) the method of concealment of allocation, (3) blinding of participants and personnel, (4) blinding of outcome assessment, (5) the completeness of outcome data, and (6) selective outcome reporting.

### 2.7. Data Synthesis and Statistical Analysis

Standard mean difference (SMD) and standard deviation (SD) or 95% confidence interval (CI) were used to evaluate the range of scores on the assessment scales between the music-based intervention and control groups. Cognitive outcomes were grouped based on the cognitive domain measured (e.g., general cognitive function, episodic memory, working memory, short-term memory, attention, processing speed, and executive function). In addition, we separately conducted meta-analyses on whether each cognitive domain included more than one study with MCI or dementia and music-based intervention by music therapist or not.

Changes from baseline values were used to conduct meta-analyses as they allowed for the comparison of more trials. When only pre- and post-data were presented, changes from baseline scores were calculated by deducting the baseline score from the follow-up score. Standard error (SE) scores were converted to standard deviation (SD) scores using the following equation [37]:

SD = SE × √N

Changes from baseline standard deviation (SD) were calculated using the following correlation coefficient equation:

SD_E/change_ = √SD^2^_E/baseline_ + SD^2^_E/final_ − (2 × 0.5 × SD_E/baseline_ × SD_E/final_)

All meta-analyses were performed to combine the effect sizes using meta packages in R [38]. If a study reported multiple change scores of cognitive tests in the same cognitive domain, then we used multilevel meta-analysis using metafor package in R [39]. Statistical heterogeneity among the trials was assessed, and a *p* value of <0.05 was regarded as statistically significant. A higher score reflecting better cognitive function was represented by a positive effect estimate. A lower score reflecting poorer task performance was represented by a negative effect estimate. A random-effects model was chosen due to the expected heterogeneity between trial protocols. Heterogeneity was measured using Higgins I^2^ statistic [40] and an I^2^ threshold of >40% was set to detect heterogeneity. Forest plots were used to graphically present the combined results.

## 3. Results

### 3.1. Literature Search and Study Selection

**Figure 1:**
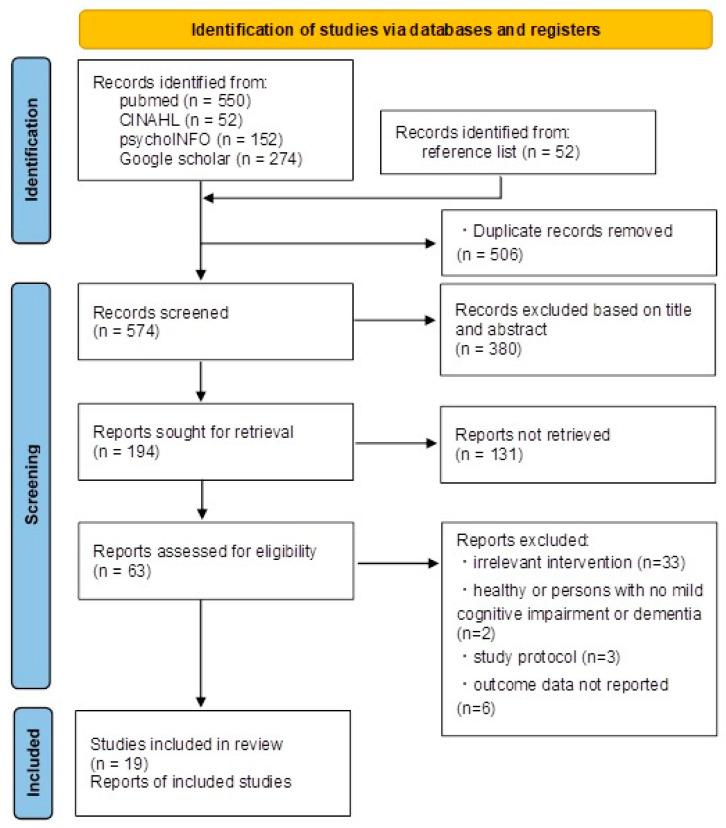
PRISMA flow chart.

A total of 522 abstracts were identified from the databases and an additional 52 potential studies were further extracted from the bibliographies of review articles. All 574 titles and abstracts were screened and the full texts of 194 studies were further evaluated for relevance. A total of 44 studies about cognitive function were excluded for the following reasons: 33 had irrelevant intervention or study groups, nine did not specify what would be required for there to be adequate data for analysis (such as a lack of standard deviation), and two compared participants with dementia and healthy controls or included only healthy adults. The definitive analysis included 19 trials for cognitive function involving participants with dementia all over the world, published between 2000 and 2021. The study selection process is presented in the PRISMA flow chart (Figure 1).

### 3.2. Characteristics of Included Studies

The 19 eligible trials were comprised of *n* = 1024 participants with a clinical diagnosis of MCI and any stage of dementia. Included RCTs were conducted in Asia (eight studies; Japan, Korea, China, and Singapore), Europe (eight studies; Italy, France, Sweden, and Belgium), and US (three studies). The sample sizes ranged from 26 to 298 (11 studies including 10–50 people; 5 studies including 51–100 people, 1 study including 101–200 people, and 2 studies including 201–300 people), the mean age ranged from 60.0 to 87.1 years, and the percentage of females ranged from 50.0% to 100% (Table 1). Regarding the severity of dementia, three studies claimed that they recruited participants with MCI [41,42,43], six studies claimed that they recruited participants with mild-to-moderate dementia [44,45,46,47,48,49], two included participants with severe dementia [50,51], and eight included participants with dementia of any severity level [45,52,53,54,55,56,57,58,59].

Regarding the type of therapy investigated, four studies investigated singing therapy [42,46,50,53], eight studies investigated music listening therapy [41,45,47,48,50,52,56,58], three studies investigated playing instruments [47,51,53], and five studies investigated music with movement therapy [43,44,53,54,59]. In five studies, the certificated music therapist provided the music sessions [44,49,52,55,60].

A total of 13 studies included comparisons with a usual care group [41,42,43,45,48,49,50,51,52,55,56,57,59] and nine studies included comparisons with an active control group [41,44,45,46,47,50,53,54,58]. The active control intervention included various therapies such as meditation [52], other art related therapies [42,45,46,47,50], or exercise [41,43], and in the blank control group, the participants received the usual care [41,42,43,45,48,49,50,51,52,55,56,57,59]. Regarding the intervention durations of the included studies, the average intervention period was 14.2 (SD = 7.5) weeks, and the average time was 51.5 (SD = 28.0) minutes (Table 1).

The included studies used a wide range of cognitive function measures and the domains are shown in Table 2.

### 3.3. General Cognitive Function

**Figure 2:**
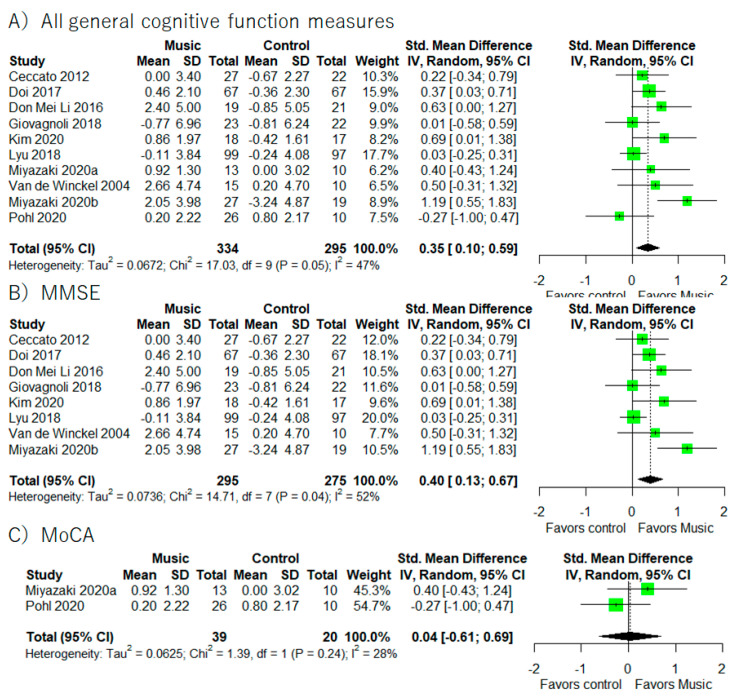
Trial level data, effect estimates, and forest plot for all general cognitive functions’ measures (**A**), MMSE (**B**), and MoCA (**C**). The area of green square is proportional to the study’s weight in the meta-analysis.

A total of 10 studies provided the results regarding general cognitive function (Figure 2) [41,42,44,50,51,54,55,56,58,59]. Of those 10 studies, eight provided results for the MMSE [41,44,50,51,54,55,56,58], two for the MoCA [42,59], and one for the SIB [52]. Meta-analysis using all studies (Figure 2) revealed that music-based intervention had a positive effect on general cognitive function (I^2^ = 47.1%, SMD = 0.35, 95% CI 0.10 to 0.59). In addition, meta-analysis on studies using only the MMSE as the outcome measure showed a significant improvement in MMSE score (I^2^ = 52.4%, SMD = 0.40, 95% CI 0.13 to 0.67).

**Figure 3:**
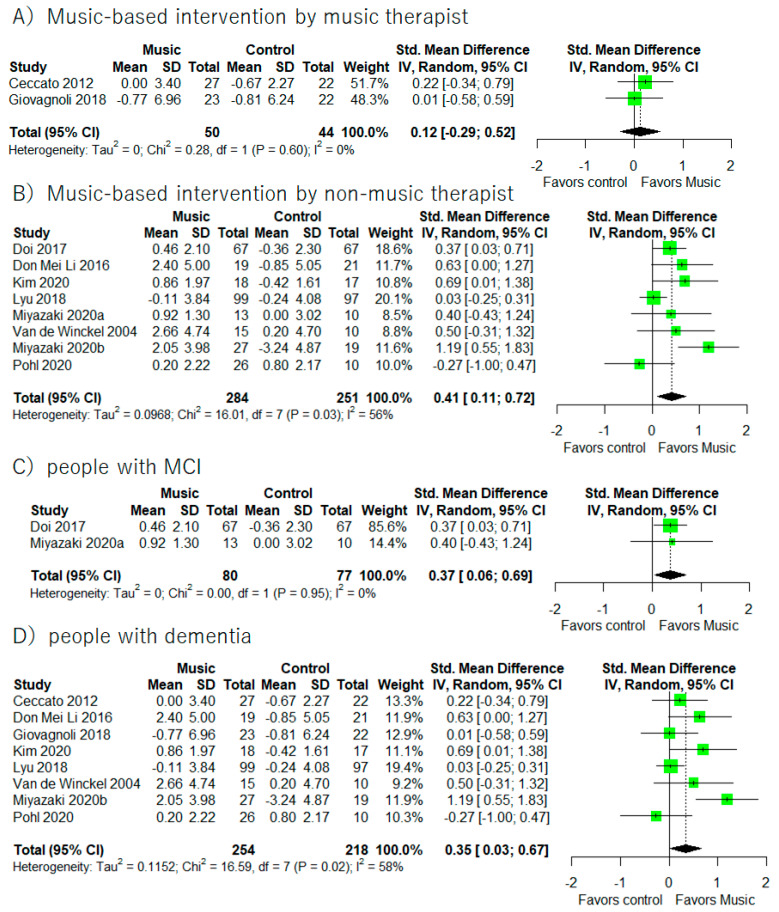
Trial level data, effect estimates, and forest plot in supplementary meta-analyses for music-based intervention by music therapist (**A**), music-based intervention by non-music therapist (**B**), people with MCI (**C**), and people with dementia (**D**). The area of green square is proportional to the study’s weight in the meta-analysis.

Supplementary Meta-analyses (Figure 3) revealed that music-based intervention improved general cognitive functions in people with MCI (I^2^ = 0%, SMD = 0.37, 95% CI 0.06 to 0.69) and dementia (I^2^ = 58%, SMD = 0.35, 95% CI 0.03 to 0.67). Moreover, music-based intervention by non-music therapists showed significant improvements in general cognitive functions (I^2^ = 56.3%, SMD = 0.41, 95% CI 0.11 to 0.72).

### 3.4. Episodic Memory

**Figure 4:**
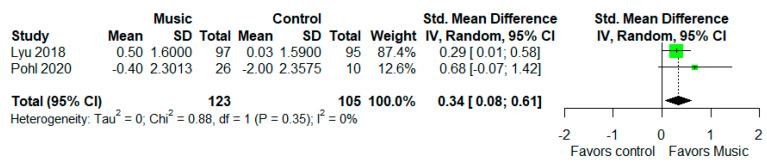
Trial level data, effect estimates, and forest plot for the effects of music-based intervention on the AVLT. The area of green square is proportional to the study’s weight in the meta-analysis.

The AVLT was used as a measure for episodic memory in two measures (Figure 4) [45,50]. Meta-analysis revealed that music-based intervention improved AVLT performance (I^2^ = 0%, SMD = 0.34, 95% CI 0.08 to 0.61).

### 3.5. Processing Speed

**Figure 5:**
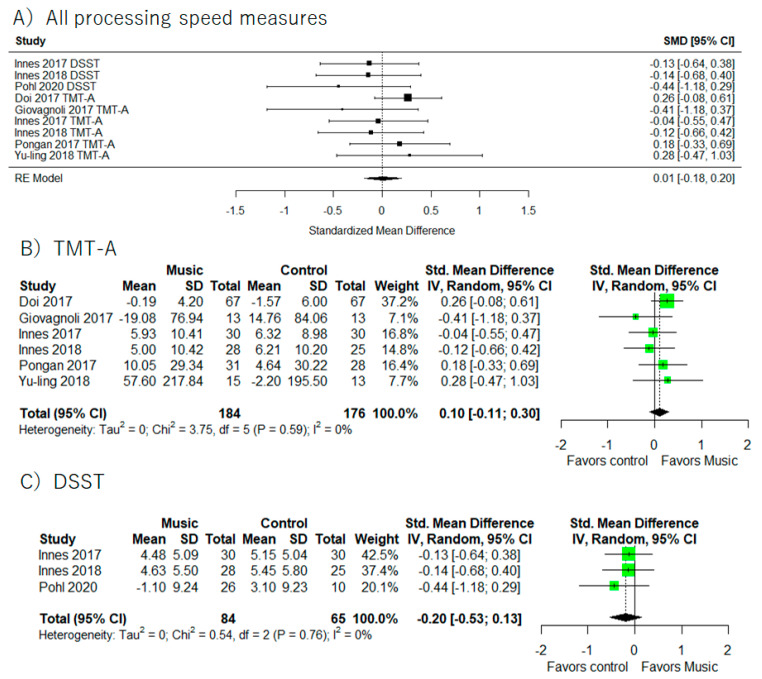
Trial level data, effect estimates, and forest plot for the effects of music-based intervention on all processing speed measure (**A**), TMT-A (**B**), and DSST (**C**). The area of green square is proportional to the study’s weight in the meta-analysis.

Processing speed results were provided in seven studies (Figure 5) [41,44,46,47,49,58,59] (I^2^ = 8.5%, SMD = 0.01, 95% CI −0.18 to 0.20). There were no statistically significant relations between music-based intervention and cognitive function. We also did not find any significant results in each cognitive test (TMT-A and DSST).

**Figure 6:**
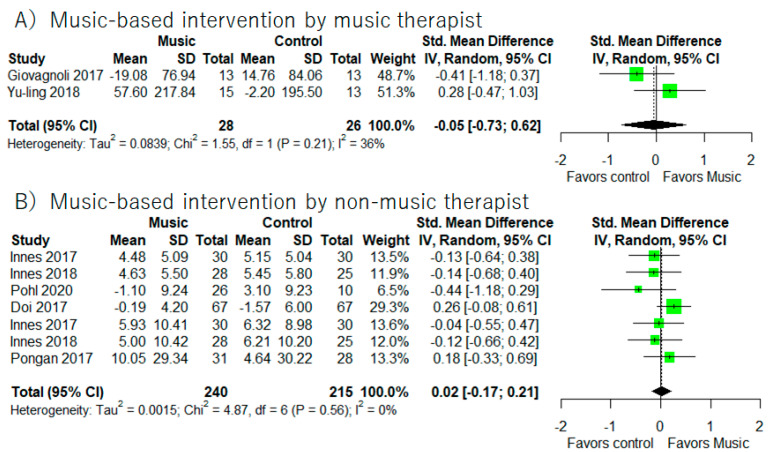
Trial level data, effect estimates, and forest plot in supplementary meta-analyses for music-based intervention by music therapist (**A**), music-based intervention by non-music therapist (**B**). The area of green square is proportional to the study’s weight in the meta-analysis.

In addition, supplemental meta-analyses (Figure 6) did not show significant improvements of processing speed based on participants (MCI or dementia) and music-based intervention by the music therapist or not.

### 3.6. Executive Function

**Figure 7:**
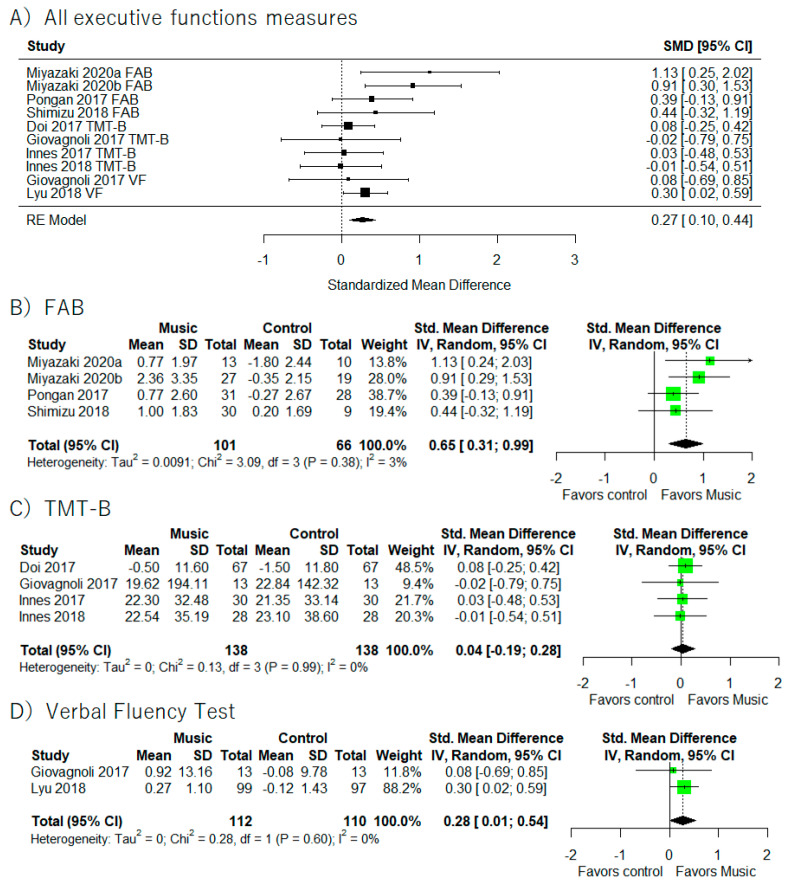
Trial level data, effect estimates, and forest plot for the effects of music-based intervention on all executive functions’ measures (**A**), TMT-B (**B**), FAB (**C**), and Verbal Fluency Test (**D**). The area of green square is proportional to the study’s weight in the meta-analysis.

A total of nine studies provided executive function results (Figure 7) [41,42,43,44,46,47,50,51,58]. Of those nine studies, four provided results for the FAB [42,43,46,51], two studies used the Verbal Fluency Test [44,50], and four used the TMT-B [41,44,47,58]. Meta-analysis using all studies revealed that music-based intervention generally improved executive function (I^2^ = 8.51%, SMD = 0.27, 95% CI 0.10 to 0.44). In addition, meta-analysis including studies that only used the FAB as the outcome measure showed the beneficial effects of music-based intervention on FAB performance (I^2^ = 3.1%, SMD = 0.65, 95% CI 0.31 to 0.99) and Verbal Fluency Test (I^2^ = 0%, SMD = 0.28, 95% CI 0.01 to 0.54).

**Figure 8:**
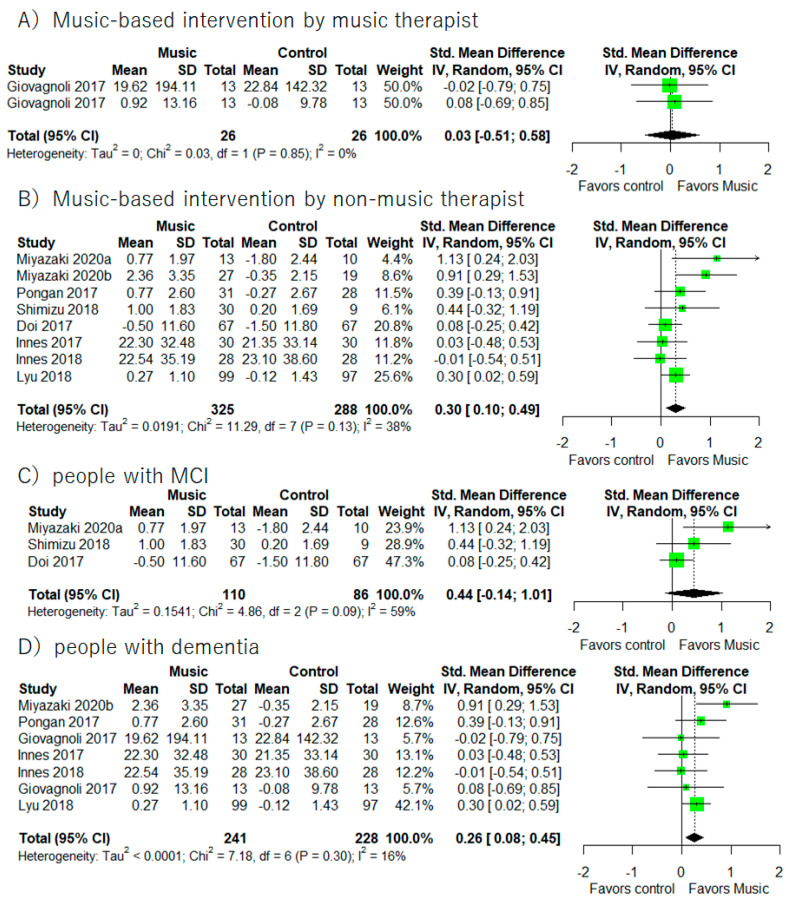
Trial level data, effect estimates, and forest plot in supplementary meta-analyses for music-based intervention by music therapist (**A**), music-based intervention by non-music therapist (**B**), people with MCI (**C**), and people with dementia (**D**). The area of green square is proportional to the study’s weight in the meta-analysis.

Supplementary meta-analyses (Figure 8) revealed that music-based intervention by non-music therapist improved executive functions (I^2^ = 38%, SMD = 0.30, 95% CI 0.10 to 0.49). In addition, music-based intervention improved executive functions in people with dementia (I^2^ = 16%, SMD = 0.26, 95% CI 0.08 to 0.45).

### 3.7. Working Memory/Short Term Memory/Attention

There were no specific studies that provided details regarding working memory, short-term memory, or attention.

### 3.8. Quality Assessment

An assessment of the methodological quality of the included studies is presented in Table 3. The quality assessment score ranged from 8 to 13, with an average of 10.47 (SD = 1.50). All included studies had sufficient methodological quality. The scores of item 5 (blinded outcome assessor), 9 (intention-to-treat analysis), and 14 (reporting CONSORT statement) were low. However, all studies fulfilled the methodological qualities of item 1 (random allocation), 4 (eligibility criteria specified), and 11 (adequate description of the control/comparison group).

## 4. Discussion

We performed a systematic review and meta-analysis to examine the effect of music-based intervention on each subdomain of cognitive function for people with dementia and MCI. We found that music-based intervention improved cognitive function assessed by the MMSE total score. Further, the intervention improved both the executive function and the episodic memory performance. These results are of key importance for patients, clinicians and stakeholders because these cognitive functions are associated with ADL and QoL in people with MCI and dementia [61,62,63].

The first main finding is that music-based intervention increased general cognitive function in people with dementia and MCI. This finding is in line with prior meta-analysis studies [17,18]. However, the results regarding the effects of music-based intervention are still controversial. Some meta-analysis studies have reported no positive effect of musical therapy on general cognitive function [19,20]. The reason for this dissociation is the number of included studies in past meta-analysis studies. For example, the number of included studies in the previous meta-analyses was eight [18], seven [19], and eight [20]. However, the current meta-analysis included 19 studies. Therefore, our meta-analysis provides more validated results.

It is important to note that most of the previous studies ignored the differences between cognitive tests. For example, the MMSE and the MoCA are mainly used to measure general cognitive function, but the sub-components of each test are different [64]. The MMSE emphasizes orientation and language activities while the MoCA emphasizes executive function and visual task domains [65]. Supporting this difference between the MMSE and the MoCA, in this meta-analysis we found that music-based intervention significantly improved MMSE scores but not MoCA scores. This result indicates that it would be better to consider this difference and separately perform meta-analysis for each test. However, we found only two studies using the MoCA [42,59]. Thus, it will be necessary to perform further meta-analysis in the near future.

Considering the types of interventions is important for an understanding of the beneficial effect of music-based intervention on cognitive function. Music-based intervention as a whole could conceptually be divided into receptive intervention type, which required the participants to listen to music, and active intervention type, which required the participants to play musical instruments, to sing songs, or to move with music. In this study, we found eight receptive interventions [41,45,47,48,50,52,56,58] and 11 active interventions [42,43,44,46,47,50,51,53,54,59]. Among the eight studies that measured MMSE scores [41,44,50,51,54,55,56,58], five studies involved receptive music activities [41,44,55,56,58] and three involved active music activities [50,51,54]. Of the eight studies, seven using the MMSE showed increases in MMSE performance [41,50,51,54,55,56,58]. These results indicate that any type of music-based intervention would have beneficial effects on general cognitive function.

The second main finding is that we demonstrated the positive effect of music-based intervention on executive function. In addition, we found that music-based intervention led to improvements in FAB and Verbal Fluency Test scores. Considering the types of interventions, three studies used receptive music activities [41,47,58] and six studies used active music activities [42,43,44,46,50,51]. Of the six studies included, the four studies using active music activities reported significant improvements in the performance of executive function [42,46,50,51]. Moreover, all four studies that measured the FAB used active music activities [42,43,46,51]. This suggests that active music-based intervention would have positive effects on executive function. This may be because active music intervention is more likely to promote socialization, engagement, verbal processing, or motor planning compared with receptive music intervention [66]. Since active music intervention has been utilized more often than receptive music intervention, future studies should investigate the effect of active music-based intervention on cognitive function.

The third and final main finding is that music-based intervention led to an improvement in episodic memory. In this meta-analysis, we included two studies [45,50], both of which used the AVLT as a measurement for episodic memory. The AVLT is an auditory verbal learning test using words [67]. The interventions asked the participants to sing and listen to their favorite songs, which were popular in their twenties and thirties, or listen to music and recall their experiences related to the music (reminiscence). The intervention required participants to understand the lyrics (semantic memory) and explicitly and implicitly recall past episodes (episodic memory) during music-based intervention. Therefore, this type of music-based intervention may facilitate episodic memory performance. Due to the small number of available studies, future studies are required that include other memory function measurements to clarify the effect of music-based intervention on episodic memory.

It would be too early to conclude that music-based intervention is not effective in processing speed performance. In this meta-analysis we found only five studies that investigated processing speed [41,44,46,47,58]. In addition, typical music-based intervention does not require the cognitive process related to processing speed [68]. Future studies should include episodic memory or processing speed components during music-based intervention.

It should be important to consider whether the intervention period of the included studies was sufficient to evaluate improvements in cognitive function. Previous systematic review and meta-analysis for non-pharmacological interventions such as cognitive training, exercise, nutrition revealed that previous studies used the similar intervention periods (from 4 to 12 weeks) [25,69,70,71,72]. The short-term intervention was able to improve cognitive functions in older adults with and without dementia. This indicated that the intervention period among the included studies was sufficient time to investigate the improvements in cognitive functions. However, whether an intervention period would affect the beneficial effect of music-based intervention on cognitive function is an important consideration. Further studies should investigate effects of shorter- and longer-term music-based intervention.

It is important to note results from supplementary meta-analyses based on MCI/dementia differences and the music-based intervention with the music therapist/the non-music-therapist (Please see Figure 3, Figure 6, and Figure 8). Music-based intervention improved general cognitive functions in both people with MCI and dementia. On the other hand, music-based intervention improved executive functions in people with dementia. Moreover, music-based intervention by the non-music therapist improved general cognitive functions and executive functions in people with MCI and dementia. However, we did not find any significant improvements from music-based intervention by the music therapist. There would be some consideration points. The main point is the small number of studies included for the supplementary analyses. We found only three studies with MCI and five studies with music therapists. The second point is the countries where the RCT was conducted. All studies with MCI were conducted in Japan. Looking at music-based intervention by a music therapist, four studies were conducted in Italy and one study was in USA. Therefore, we were unable to exclude the possibility of a cultural bias. In the future, there should be more studies to investigate whether benefits from music-based intervention would differ between people with MCI and dementia and between music-based intervention by a music therapist or not.

In the current meta-analysis, we did not include music-based interventions transduced into vibrotactile stimulation or single frequency sound. The music interventions we reviewed are those with sound perceived culturally as music and which we can naturally hear (20 Hz–20 kHz). Although vibrotactile aspects of sound and vibratory dimensions of music are frequently present as potentially therapeutic effects, they are less studied. A recent scoping review reported that vibration intervention had positive effects on cognitive functions [73]. In the future study, we should consider beneficial effects of music-based intervention using vibration on cognitive functions.

This study has several limitations. First, clinical heterogeneity between studies, such as severity of dementia, could not be avoided. Second, although we separately analyzed the clinical cognitive function assessment scales, some of the outcome domains had only one study. Third, some of the included studies had a small sample size. Due to the small number of included studies and the wide variety of participant characteristics, we were unable to conduct a stratified analysis. Fourth, the placebo effect in music intervention studies should have been mentioned. Participants did not know the purpose of the music intervention because they were blinded in 15 of the 19 included studies (see Table 1). However, almost all studies used passive control groups. In this case, participants in the music intervention group would think that they receive a beneficial intervention/treatment. Therefore, a placebo effect might occur because they would expect that their cognitive function would be improved. To reduce the placebo effect, the future study should use an active control intervention group. Finally, we did not consider a kinesthetic and physical aspect in the music-based intervention program. To exclude an effect of physical activity, in the future, we should compare similar physical activities groups with and without music.

## 5. Conclusions

This meta-analysis illustrates that music-based intervention can improve general cognitive function, executive function, and episodic memory performance in older adults with MCI and dementia. Music-based intervention, compared with other non-pharmacological interventions, is more implementable and less costly with little chance of adverse effects. Therefore, music-based intervention appears to be a suitable intervention to apply more frequently in the clinical field.

## Figures and Tables

**Table 1:** Characteristics of included randomized controlled trials.

Study and Year	Country	Recruitment Site	No. of Participants	Mean Age	Female	Severity of Dementia	Type of Music-Based Intervention	Control Type	Intervention Period	Intervention Frequency	Time for Each Intervention	Study Outcomes	Quality Score
Ceccato 2012 [55]	Italy	Support center	51	86.3	40 (78.4%)	Non-specific	STAM (music with movement, clapping hands)	Control	12 weeks	Twice a week	40 min	MMSE	10
Yu-ling 2018 [49]	USA	Dementia outpatient unit at a medical center	28	77.3	14 (50%)	Mild to moderate	Musical dual task (sing or play instrument while walking)	Control	8 weeks	Once a week	60 min	MMSE, TMT-A	12
Doi 2017 [41]	Japan	National center for geriatrics and gerontology	201	76	104 (51.7%)	MCI	Play instrument (percussion)	Dance, control	40 weeks	Once a week	60 min	MMSE, TMT-A, TMT-B, Story and word memory	11
Don Mei Li 2016 [56]	China	Long term care facility	40	82.4	28 (70%)	Non-specific	Folk recreation (singing)	Control	16 weeks	3 times a week	40–50 min	TMT-A	8
Giovagnoli 2017 [44]	Italy	One nursing center	39	73.6	24 (61.5%)	Mild to moderate	Play instrument	Active control (cognitive training, neuroeducation)	12 weeks	Twice a week	45 min	TMT, DSST	9
Giovagnoli 2018 [52]	Italy	One center	45	73.2	31 (68.9%)	Non-specific	Music (playing music) and drugs	Drugs only	24 weeks	Twice a week	40 min	SBI, MMSE	13
Innes 2017 [58]	USA	Community	60	60.6	56 (93.3%)	Non-specific	Listening to music	Meditation	12 weeks	Daily	12 min	TMT-A, TMT-B, DSST,	12
Innes 2018 [47]	USA	Community	53	60	46 (86.8%)	Non-specific	Listening to music	Meditation	12 weeks	Daily	30–45 min	TMT-A, TMT-B	11
Kim 2020 [48]	Korea	Adult day care center	35	79.3	26 (74.3%)	Mild AD	Recollection based occupational music-based intervention (singing, listening)	Control	24 weeks	5 sessions a week	60 min	MMSE	9
Lyu 2018 [50]	China	Geriatric hospital	298	69.7	173 (58.1%)	Mild to severe AD	Singing	Lyric reading, control	12 weeks	Twice a day, twice a week	30–40 min,	Verbal fluency, AVLT, MMSE.	10
Mahendran 2018 [45]	Singapore	Community living	68	71.1	38 (55.9%)	Mild neurocognitive decline	Music reminiscence (listening)	Art therapy, control	12 weeks	Once a week	65 min (including 15 min break)	AVLT	11
Miyazaki 2020a [42]	Japan	Residential care facilities	26	81.5	20 (76.9%)	MCI	Singing (karaoke)	Active control	12 weeks	Once a week	120 min	MoCA, FAB	12
Miyazaki 2020b [51]	Japan	Nursing home	46	87	40 (87.0%)	Non-specific	Play instrument (Drum)	Control	12 weeks	Twice a week	30 min	MMSE, FAB	9
Narme 2013 [53]	France	Nursing home	37	87.1	32 (86.5%)	Alzheimer’s with mixed dementia	Play instrument (Percussion)	Cooking	4 weeks	Twice a week	60 min	SBI	8
Pohl 2020 [59]	Sweden	Community dwelling individuals diagnosed with PD	46	70	32 (69.6%)	PD	Training with music based intervention	Control	12 weeks	Twice a week	60 min	MoCA, PDQ39	11
Pongan 2017 [46]	France	University hospital	59	79.5	39 (66.1%)	Mild Alzheimer’s disease	Singing	Painting	12 weeks	Once a week	120 min	TMT-A, FAB,	13
Raglio 2015 [60]	Italy	Nursing home and day care centers	120	81.7	94 (78.3%)	Moderate to severe dementia	Active music therapy, listening to music	Control	10 weeks	Twice a week	30 min	MMSE	11
Shimizu 2018 [43]	Japan	Community dwelling individuals participating in the dementia care class	45	74.6	38 (84.4%)	MCI	Music with movement	Active control (movement without music)	12 weeks	Once a week	60 min	FAB	10
Van de Winckel 2002 [54]	Belgium	Public hospital	25	81.7	25 (100%)	Non-specific	Exercise with music	Active control (one to one conversation)	12 weeks	Once a day	30 min	MMSE, ADS 6	9
Total			1024										

Note: MMSE scores: 0~11 severe dementia, 12~17 moderate dementia, 18~23 mild dementia, 23~30 no dementia.

**Table 2:** Description of clinical assessment scale in different domains.

Outcome	Test	Number of Studies	Number of Studies Compared with Control Group	Number of Studies Compared with Active Control Group
General cognitive function	MMSE	8	5	3
	MoCA	2	1	1
Episodic memory	AVLT	2	2	2
Working memory		0	-	-
Short-term memory		1	-	-
Attention		0	-	-
Processing speed	TMT-A	6	2	6
	DSST	1	0	1
Executive function	Verbal fluency	2	2	2
	TMT-B	4	0	4
	FAB	5	2	3

**Table 3:** Quality assessment scores of included studies using modified Delphi list.

Lead Author, Year, Country	Q1	Q2	Q3	Q4	Q5	Q6	Q7	Q8	Q9	Q10	Q11	Q12	Q13	Q14	Total
Ceccato 2012 [55]	Y	Y	Y	Y	?	?	Y	Y	N	Y	Y	Y	Y	N	10
Yu-ling 2018 [49]	Y	Y	N	Y	Y	?	Y	Y	Y	Y	Y	Y	Y	Y	12
Doi 2017 [41]	Y	Y	Y	Y	Y	?	Y	Y	Y	Y	Y	N	Y	N	11
Don Mei Li 2016 [56]	Y	Y	Y	Y	?	?	Y	Y	N	N	Y	N	Y	N	8
Giovagnoli 2017 [44]	Y	Y	Y	Y	?	N	?	Y	N	Y	Y	Y	Y	N	9
Giovagnoli 2018 [52]	Y	Y	Y	Y	Y	?	Y	Y	Y	Y	Y	Y	Y	Y	13
Innes 2017 [58]	Y	Y	Y	Y	?	Y	Y	Y	Y	Y	Y	Y	Y	N	12
Innes 2018 [47]	Y	Y	Y	Y	Y	?	Y	Y	N	Y	Y	Y	Y	N	11
Kim 2020 [48]	Y	Y	Y	Y	?	?	Y	Y	N	N	Y	Y	Y	N	9
Lyu 2018 [50]	Y	Y	Y	Y	Y	N	N	Y	N	Y	Y	Y	Y	N	10
Mahendran 2018 [45]	Y	Y	Y	Y	?	N	Y	Y	Y	N	Y	Y	Y	Y	11
Miyazaki 2020a [42]	Y	Y	Y	Y	Y	Y	Y	?	N	Y	Y	Y	Y	Y	12
Miyazaki 2020b [51]	Y	N	Y	Y	N	N	N	Y	N	Y	Y	Y	Y	Y	9
Narme 2013 [53]	Y	Y	Y	Y	?	?	?	Y	N	N	Y	Y	Y	N	8
Pohl 2020 [59]	Y	Y	Y	Y	N	N	Y	Y	N	Y	Y	Y	Y	Y	11
Pongan 2017 [46]	Y	Y	Y	Y	Y	Y	Y	Y	Y	Y	Y	Y	Y	?	13
Raglio 2015 [60]	Y	Y	Y	Y	?	?	Y	Y	Y	Y	Y	Y	Y	N	11
Shimizu 2018 [43]	Y	Y	Y	Y	N	?	Y	Y	N	Y	Y	Y	Y	N	10
Van de Winckel 2002 [54]	Y	Y	Y	Y	?	?	Y	Y	N	N	Y	Y	Y	N	9
Total score across studies	19	18	18	19	7	3	15	18	7	14	19	17	19	6	-

Note: (Q1) Random allocation, (Q2) treatment allocation concealed, (Q3) groups/subjects similar at baseline regarding important prognostic values, (Q4) eligibility criteria specified, (Q5) blinded outcome assessor, (Q6) blinded care provider, (Q7) blinded patient, (Q8) point estimates and measures of variability presented for the primary outcome measures, (Q9) intention-to-treat analysis, (Q10) details of random allocation methods, (Q11) adequate description of the control/comparison group, (Q12) between-group statistical comparison, (Q13) reporting dropouts, (Q14) reporting CONSORT statement. Y means that previous studies met the criteria of each item. N means that previous studies did not met the criteria of each item. ? means that previous studies did not mention the item.

## Data Availability

The datasets used and analyzed in the current study are available from the corresponding author on reasonable request.

## Supplementary Materials

The following supporting information can be downloaded at: https://www.mdpi.com/article/10.3390/healthcare10081462/s1, Table S1: PRISMA checklist.
